# The Occurrence of Stress, Illness Acceptance and the Quality of Life of Patients after Pacemaker Implantation

**DOI:** 10.3390/ijerph192114133

**Published:** 2022-10-29

**Authors:** Kamil Sikora, Agnieszka Wawryniuk, Robert Jan Łuczyk, Katarzyna Sawicka, Agnieszka Zwolak

**Affiliations:** Department of Internal Medicine and Internal Nursing, Chair of Preventive Nursing, Faculty of Health Sciences, Medical University of Lublin, Ul. Chodźki 7, 20-093 Lublin, Poland

**Keywords:** quality of life, psychological stress, artificial pacemaker, behavior

## Abstract

Pacemaker implantation improves the quality of life of most patients, especially in the initial period after implantation. It is necessary to measure the long-term quality of life and factors that can affect it—stress and illness acceptance. The aim of the study was to assess the impact of stress and illness acceptance on the quality of life of patients after pacemaker implantation. To obtain final conclusions, we performed a survey on a group of 100 patients with implanted pacemakers. Our survey consists of standardized research tools to check the quality of life (WHOQOL-BREF), perceived stress and ways to cope with it (PSS-10, mini-COPE) and acceptance of illness (AIS). The results of the study were summarized in a statistical analysis. At least a good quality of life was declared by more than half of the respondents [Me = 4; 95% PU (4, 4)]. The average result obtained by the respondents when converted to the STEN scale was six. The respondents were characterized by a moderate level of stress compared to the PSS-10 norms and it was related to the quality of life. Similar, statistically significant correlations were presented as mini-COPE and AIS results. Respondents were most likely to use acceptance strategies, active coping methods, when dealing with something else and planning. The rarest strategies were doing nothing and taking pharmaceuticals. The average score on the acceptance of illness scale was (M = 22.14; SD = 6.05), which is more than the result obtained by patients from the AIS normalization group. It shows that assessed patients after pacemaker implantation declare the general quality of life as good or higher. Additionally, this quality of life is closely related to stress levels, coping strategies and acceptance of illness, which shows us the importance of research in this area.

## 1. Introduction

According to 2017 data provided by the National Health Fund, approximately 30,000 pacemakers and 10,000 cardioverter-defibrillators have been implanted in Poland, whereby pacemaker implantation improves patients’ quality of life, which we can observe in the literature review. Despite this, there is still a lack of research in the area of quality of life after pacemaker implantation [1,2,3].

The beginning of research based on the quality-of-life topic began in the 1960s. Despite the passage of years, in the last two decades, this concept has gained wider interest. Initially, it was thought that a high quality of life could be achieved if you had valuable material goods; however, the research conducted overturned this view and resulted in an increase in the interest of scientists [4]. Therefore, the quality of life is inevitably related to the activities of researchers dealing with issues related to human existence. We can observe it in economic and philosophical research, as well as in the fields of sociology and medicine. Despite the similarity of the topic being the research area, quality of life will be understood differently in economics, differently in psychology and still differently in medical sciences or health sciences [5,6]. Despite the diversity of definitions and problems in defining what the quality of life is, there is no terminology that combines all criteria and elements, is not controversial and can be used in any field of knowledge [7].

Health-related quality of life (HRQoL) is a cornerstone in medical science. It provides reliable information on the impact of a disease entity or the patient’s clinical condition on both subjective and objective disease symptoms, allowing it to be documented and describing changes in the course of the disease over the years. In addition, it can be useful in the diagnostic process, prognosis assessment or planning care for patients. The assessment of HRQoL and the criteria affecting it is therefore of great importance in improving the quality of medical care for the entire community [8].

Researchers regard that some psychological determinants could have a big importance in general health conditions, especially in cardio-metabolic pathways. There are many factors, which can affect the patient’s HRQoL. Of course, one of the most important factors is the general patient condition and physical well-being. However, on the other hand, we have obtained mental factors like the occurrence of stress, anxiety and illness acceptance, which were the subject of our study [9]. The quality of life should not be assessed only in a few domains. A total assessment of a patient’s quality of life takes into account not only determinants in the form of health status and correctness of treatment but also the patient’s ability to cope with problems, the severity of stress and the ability to accept disease [10]. The aim of the study was to assess the impact of stress and illness acceptance on the quality of life of patients after pacemaker implantation. To achieve it, we used standardized research tools to perform a survey on a group of 100 patients after pacemaker implantation.

## 2. Materials and Methods

The survey was conducted between January and May 2020. Respondents were patients with implanted pacemakers, treated in the cardiological ward of Independent Public Clinic Hospital No. 4 in Lublin, Poland. A sample of 100 patients post-pacemaker implantation give consent to take part in the study.

Before performing the study, we receive the consent of the respondents, head of hospital organizational units and bioethics committee. We collected sociodemographic data—age, sex, place of living and education. The study was conducted by diagnostic survey method by using the following standardized research surveys to assess general quality of life, stress level and ways to cope with it and illness acceptance as follows:

World Health Organization Quality of Life (WHOQOL-BREF)—international questionnaire developed and translated to 50 languages by WHOQOL group from World Health Organization. It is a shorter version of WHOQOL-100 questionnaire. The tool was validated for Polish conditions by K. Jaracz et al. for psychometric assessment of the quality of life of healthy and sick people. Cronbach’s α coefficient is >0.7 in all domains except social domain. The questionnaire contains 26 closed-ended single-choice questions, and assessment is made in 4 domains—somatic, psychological, social and environmental, and separately for overall quality of life and health self-assessment. Final result is based on self-assessment on a scale from 1 to 5 in questions adequate for every domain [11].

Perceived Stress Scale (PSS-10)—tool by authors S. Cohen, T. Kamarc and R. Mermelstein, Polish adaptation by Z. Juczyński and Nina Ogińska-Bulik. Cronbach’s alpha coefficient for tool validity is 0.86. The reliability established by testing a group of 30 students twice at an interval of 2 days was 0.90 and at an interval of 4 weeks was 0.72. It consists of 10 questions assessing the frequency of events and problems, on a scale of 0-never, 1—almost never, 2—sometimes, 3—fairly often and 4—very often. The total score, reflecting the level of perceived stress, is the sum of the points obtained, expressed on the STEN scale, where scores from 1 to 4 are low, from 5 to 6 as average and above 7 STEN as high intensity of stress [12,13].

Acceptance of Illness Scale (AIS)—survey developed by Felton, Revenson and Hinrichsen, adapted to Polish conditions by Z. Juczyński. The Cronbach’s alpha internal consistency coefficient was 0.85. The validity of AIS was tested by comparing the results of the Scale with assessment of oncological patients’ treatment effects. Significant correlation was obtained (0.42; *p* < 0.01). It is used to assess the level of illness acceptance among ill people. This scale contains 8 one-sentence statements expressing a negative attitude to the illness. Subjective agreement with the described sentences should be expressed on a scale from 1 to 5, where 1—means strongly agree and 5—strongly disagree. The sum of points reflects the level of illness acceptance, with a score up to 18—as low illness acceptance, ranging from 19 to 29—as average acceptance, and above 30 as total illness acceptance [14,15].

Coping Orientation to Problems Experienced questionnaire (Mini-COPE)—shortened version of the COPE Multidimensional Inventory. Original version of questionnaire was created by Charles S. Carver. The Polish adaptation by Z. Juczyński and N. Ogińska-Bulik has reliability of 0.86 and it was validated by correlating Mini-COPE scores with the Mini-MAC scale, designed to measure coping strategies and by predicting the severity of posttraumatic stress symptoms in a group of mothers of children treated for leukemia. Mini-COPE contains 28 statements, where every two statements correspond to a selected strategy for coping with stress. The statements are to relate to the different respondent behaviors in a difficult life situation. The frequency of behaviors should be placed on a scale of 0—I hardly ever do this, 1—I rarely do this, 2—I often do this, 3—I almost always do this. Results allow for assigning behaviors to given strategies, i.e., active coping, sense of humor, seeking emotional support, planning, positive revaluation, acceptance, turning to religion, seeking instrumental support, denial, taking in psychopharmaceuticals, dealing with other things, giving vent to one’s feelings, doing nothing or blaming yourself [12,16].

The results of the study were summarized in a statistical analysis by using SPSS Statistics ver. 25. We assumed *p*-value at *p* < 0.05 as statistically significant. To describe final data contributions we use mean, median, standard deviation, minimum and maximum values, as well as the Shapiro–Wilk test to assess distribution. The results were visualized using histograms and box plots. The analysis of the relations between variables was performed using Spearman’s rho coefficient.

## 3. Results

The average age of the respondents was 56.61, with a standard deviation of 15.89. The youngest respondent was 24 years old and the oldest was 91. Men represented 43% of the respondents, while women represented 57%. The majority of the respondents came from the city (62%), while 38% of the respondents lived in the countryside. In total, 37% of the respondents had secondary education, 30% had higher education, 24% had vocational education and 9% had primary education.

### 3.1. Overall Quality of Life of the Respondents

The general level of quality of life of the respondents, measured with the standardized WHOQOL—BREF survey, is presented below.

**Figure 1:**
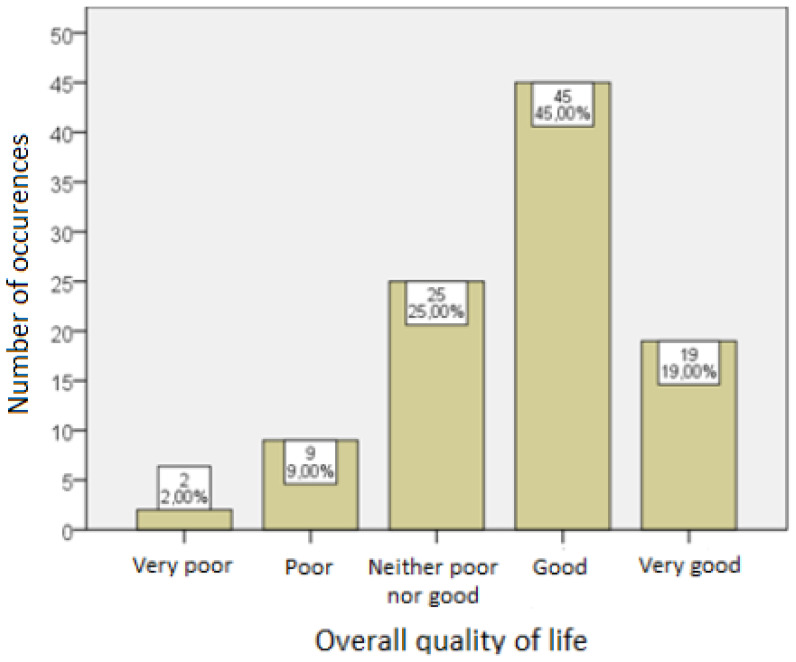
Overall quality of life of the respondents.

Figure 1 shows the distribution of answers to the question about the overall quality of life of the respondents.

At least a good quality of life was declared by more than half of the respondents [Me = 4; 95% PU (4, 4)]. The average score was 4.00 [95% CI (3.83, 4.17)] with a standard deviation of 0.71.

### 3.2. Stress Severity, Coping Strategies, Illness Acceptance and the Quality of Life of the Respondents

Below are presented the following results of the analysis of the relations between the respondents’ quality of life, their level of perceived stress, coping strategies and illness acceptance.

**Figure 2:**
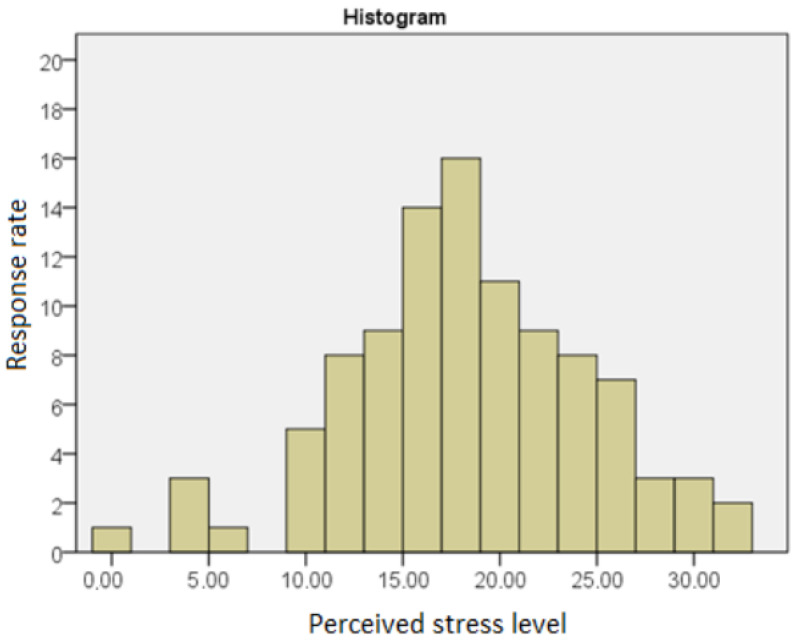
The level of perceived stress of the respondents.

Figure 2 presents the distribution of the results obtained by the respondents on the PSS—10 scale.

The average score on the perceived stress scale obtained by the respondents was 17.86, with a standard deviation of 6.12. The performed Shapiro–Wilk test showed no statistically significant deviations of the obtained distribution from results from the normal distribution (SW = 0.986; *p* = 0.348).

The average result obtained by the respondents when converted to the STEN scale was six, with a standard deviation of two. The respondents were characterized by a moderate level of stress compared to the PSS-10 norms.

Table 1 presents statistics describing the distributions of the scores obtained on each scale of the mini-COPE questionnaire.

Respondents were most likely to use acceptance strategies, active coping methods, when dealing with something else or planning. The rarest strategies were doing nothing and taking pharmaceuticals.

The Shapiro–Wilk tests showed that the distributions of all the obtained results differed statistically significantly from the normal distribution. In the case of taking pharmaceuticals, there was a clear asymmetry in the distribution of the obtained results, characterized by the prevalence of relatively low results. In the strategy of acceptance, there was a clear concentration of results around the mean value (the group was homogeneous).

Table 2 presents the results of the analysis of the relations between respondents’ quality of life, level of perceived stress, illness acceptance and stress coping strategies.

The conducted analysis showed statistically significant, moderate relations between the level of stress experienced by the respondents and their quality of life in all its domains, except for the self-assessment of health, where this relation was moderate.

The analysis also showed relations between strategies for coping with stress and the quality of life of the respondents.

Respondents had a higher overall quality of life if they more often focused on active coping, planning, positive re-evaluation, acceptance and less often if they blamed themselves and if they did nothing.

Respondents’ self-assessment of health was higher if they more often coped with stress actively, planned, re-evaluated and accepted situations, and less often if they did nothing and blamed themselves.

Respondents had a higher assessment of functioning in the physical domain if more often they dealt with stress actively, planning, re-evaluating situations positively and responding to them with humor, and less often if they ceased activities and blamed themselves.

Respondents had a higher assessment of functioning in the psychological domain more often if they dealt with stress actively, planning, re-evaluating situations in a positive way, accepting it and responding to it with humor, and less often if they stopped activities, denied and blamed themselves.

Respondents had a higher assessment of functioning in the social domain if they more often dealt with stress actively, planning, re-evaluating situations in a positive way, looking for emotional support and responding to it with humor, and less often if they stopped acting, denying and blaming themselves.

Respondents had a higher assessment of functioning in the environmental field if more often they dealt with stress actively, planning, re-evaluating situations positively, accepting it, they looked for emotional support and less often if they stopped acting, denying, focused on discharging emotions and blaming themselves.

Conducted analyses showed statistically significant relations between perceived stress and the quality of life. The higher level of perceived stress was presented by the respondents if they had a lower assessment of the quality of life.

Conducted analyses showed that the lower perceived stress of the respondents was related to their higher self-assessment of the quality of life in the domains of physical, psychological, social and environmental.

Conducted analyses also showed statistically significant relations between the level of acceptance of illness by the respondents and all domains of the quality of life, as well as its overall assessment. All correlations were at least moderate.

If higher the acceptance of illness respondents had, the higher their overall quality of life and self-assessment of health condition. The relations between the variables were moderate.

Additional analysis were carried out to determine the relations between the strategies for coping with stress and the quality of life of the respondents by controlling the level of perceived stress. For this purpose, a rank analysis of partial correlations was used. Table 3 below presents the relations between perceived stress and the choice of individual coping strategies by the respondents.

The higher level of perceived stress the respondents had, if more often they reacted to it by dealing with something else, unloading emotions, denying and blaming themselves, while less often if they reacted to it by planning, with a sense of humor, actively coping with it, positively re-evaluating, looking for emotional support and accepting the situation.

The following results of the analysis of the relations between the quality of life and illness acceptance in a group of patients with an implanted pacemaker are presented below.

**Figure 3:**
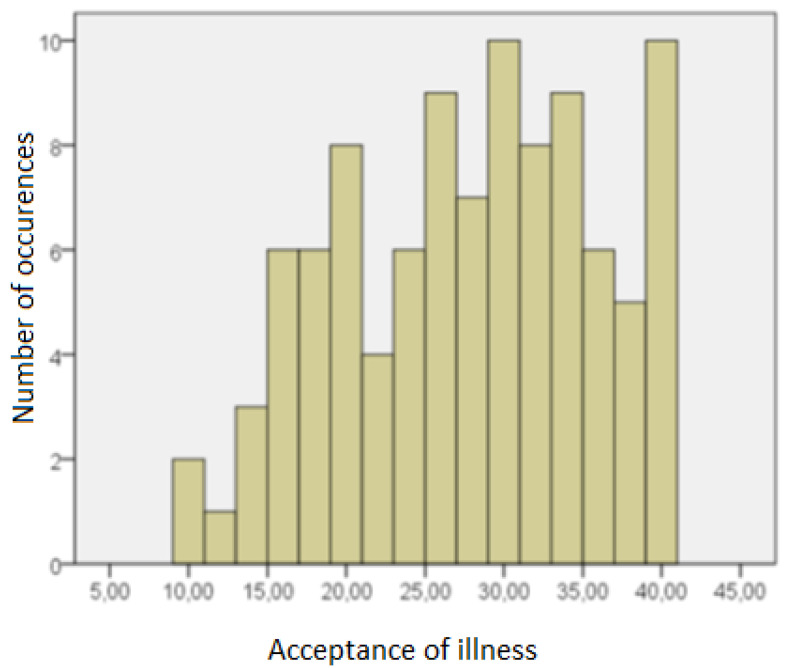
Respondents results in acceptance of illness scale.

Figure 3 presents results obtained by the respondents on the acceptance of illness scale.

The average level of illness acceptance in the group of respondents was 27.23, with a standard deviation of 8.18. The Shapiro–Wilk test showed that it differed statistically significantly from the normal distribution (SW = 0.964, *p* = 0.008).

The average score obtained by the respondents on the AIS scale differed from the average score of people from the AIS scale normalizing group who had suffered from myocardial infarction (M = 22.14; SD = 6.05), t (140) = 3.634; *p* < 0.001. The respondents had a higher sense of the quality of life than people after a heart attack.

If respondents accepted illness more, the more satisfied they were with the environment of functioning, social relations and their own physical and psychological functioning. The relations between the variables were moderate, except for a strong correlation between physical functioning and acceptance of the disease.

## 4. Discussion

The assessment of the perceived stress among the examined patients showed a moderate level of stress compared to the PSS-10 standards. The obtained result was 6 ± 2 on the STEN scale. In order to extend the research, the level of severity of stress in patients with pacemakers should be compared to the population of healthy people. Research by A. Bejer and R. Bieniek on stress and the occurrence of cardiovascular diseases did not show greater exposure to stress during illness [17]. However, in our own research, stress’s impact on the quality of life was observed in all its domains. Among the selected strategies for coping with stress, those with a positive character, such as active coping, planning or acceptance, prevailed. The smallest percentage of respondents declared coping with stress by taking pharmaceuticals or psychoactive substances—most often with a coexisting high level of stress. Similar dependencies were noticed by M. Kózka, also inside a group of patients treated in the cardiology department [18]. People that chose adaptive strategies, generally recognized as correct, i.e., actively seeking solutions, planning and making positive revaluations, also had a higher overall quality of life assessment than patients that were passive or using repression mechanisms. Conclusions in this aspect are similar to those observed by M. Mroczkowska [19]. Research conducted by Guan T. et al. on a group of 263 patients with prostate cancer shows a potential quality of life improvement after choosing adaptive coping strategies and rejecting avoidance and denial strategies in this group of patients [20]. Not only is the choice of coping strategies perceived objectively as good important for the quality of life. Conclusions from Kristofferzon ML et al. study show that sometimes patients have a better quality of life if they choose fewer coping strategies, but only self-selected [21].

The acceptance of illness, measured with the AIS scale, was also adequate for the quality of life of the respondents. The high level of the declared quality of life in each domain was closely related to the illness acceptance level (*p* = 0.000 in all domains) and this correlation was stronger than the correlation between the quality of life, perceived stress level and coping strategies. It shows us that acceptance of illness can be closely dependent on the quality of life in these groups of patients. The acceptance level was higher than an inside group of people after myocardial infarction from the standardization group. In our own research, a higher level of disease acceptance and a similar, positive correlation with all domains of the quality of life were obtained, as in the research conducted by D. Kurpas [22,23]. The impact of acceptance of illness on health-related quality of life could be found for other groups of patients with cardiac diseases. A study conducted by Jankowska-Polańska B. and coauthors presents similar results to results obtained in their own research. Patients with higher acceptance of illness have obtained a higher quality of life, which shows us the importance of assessing acceptance of illness [24].

The assessment of the quality of life in the conducted own research, due to its multi-stage and multi-dimensional nature, can be used to improve the quality of care for patients after pacemaker implantation. The influence of individual factors—stress, acceptance of the disease, severity of anxiety and depression can be eliminated or at least reduced with the use of psychological support and cardiac rehabilitation, starting from the period before pacemaker implantation. The problems outlined in these studies provide a picture of the deficits occurring in this group of patients and their level of adaptation, and may be the starting point for diagnosis and care planning, not only by specialists in the field of nursing or medicine [25,26,27].

## 5. Conclusions

The overall assessment of the quality of life shows that the quality of life is at least good in the majority of surveyed patients after pacemaker implantation. The intensity of stress in a group of surveyed patients with an implanted pacemaker is moderate. The occurrence of high levels of stress is accompanied by a low quality of life. The high quality of life of surveyed people with pacemakers is associated with a high level of illness acceptance, which is characteristic of most patients.

The selection of strategies for coping with stress, generally defined as normal, is a characteristic for surveyed patients with high quality of life.

This conclusion shows the importance of monitoring some physiological aspects during chronic disease. Therefore, despite the lack of standardization and difficulties in measurement, it seems necessary to conduct further studios, especially on a bigger group of patients, with other chronic diseases, by using other methodological methods to assess the quality of life and each factor that can have an influence on it.

## Figures and Tables

**Table 1:** The result of stress coping strategies chosen by respondents.

Variable	Results						Converted Results (STEN Score)	
	M.	SD	min	max	SW	p	M.	SD
Active stress coping methods	2.02	0.70	0.00	3.00	0.917	0.000	6	2
Planning	2.00	0.63	0.50	3.00	0.927	0.000	6	2
Seeking instrumental support	1.74	0.74	0.00	3.00	0.945	0.000	6	2
Seeking emotional support	1.93	0.78	0.00	3.00	0.928	0.000	6	2
Self-blaming	1.41	0.80	0.00	3.00	0.943	0.000	6	2
Turning to religion	1.60	1.03	0.00	3.00	0.891	0.000	7	2
Positive re-evaluation	1.94	0.72	0.00	3.00	0.919	0.000	6	2
Giving vent to one’s feeling	1.49	0.64	0.00	3.00	0.940	0.000	7	2
Acceptance	2.21	0.60	0.00	3.00	0.858	0.000	7	2
Denial	1.16	0.77	0.00	3.00	0.931	0.000	7	2
Dealing with other things	2.07	0.75	0.00	3.00	0.913	0.000	7	2
Doing nothing	0.84	0.65	0.00	2.50	0.906	0.000	7	2
Taking in pharmaceuticals	0.49	0.77	0.00	3.00	0.667	0.000	6	2
Sense of humor	1.14	0.75	0.00	3.00	0.937	0.000	6	2

M—average; Me—median; SD—standard deviation; SW—Shapiro–Wilk test result; p—test probability.

**Table 2:** Relation between respondents’ quality of life, perceived stress level, stress coping strategies and acceptance of illness.

Variable	Value	Physical Domain	Psychological Domain	Domain of Social Relations	Environmental Domain	Overall Quality of Life	Self-Assessment of Health Condition
Active stress coping methods	rho	0.489	0.533	0.434	0.482	0.495	0.389
	p	0.000	0.000	0.000	0.000	0.000	0.000
Planning	rho	0.497	0.461	0.495	0.340	0.434	0.397
	p	0.000	0.000	0.000	0.001	0.000	0.000
Seeking instrumental support	rho	0.116	0.097	0.187	0.144	0.122	0.104
	p	0.250	0.338	0.063	0.152	0.226	0.302
Seeking instrumental support	rho	0.123	0.191	0.317	0.302	0.104	0.050
	p	0.223	0.057	0.001	0.002	0.305	0.622
Self-blaming	rho	−0.243	−0.364	−0.188	−0.162	−0.261	−0.338
	p	0.015	0.000	0.060	0.107	0.009	0.001
Turning to religion	rho	−0.129	−0.181	−0.032	−0.060	−0.070	−0.086
	p	0.200	0.072	0.752	0.554	0.487	0.397
Positive re-evaluation	rho	0.390	0.480	0.348	0.266	0.414	0.337
	p	0.000	0.000	0.000	0.008	0.000	0.001
Giving vent to one’s feeling	rho	−0.189	−0.180	−0.180	−0.207	−0.190	−0.109
	p	0.060	0.073	0.072	0.039	0.058	0.282
Acceptance	rho	0.177	0.392	0.117	0.231	0.322	0.297
	p	0.078	0.000	0.245	0.021	0.001	0.003
Denial	rho	−0.175	−0.319	−0.224	−0.335	−0.093	−0.076
	p	0.082	0.001	0.025	0.001	0.357	0.451
Dealing with other things	rho	0.179	0.139	0.167	0.113	0.183	0.162
	p	0.075	0.167	0.097	0.264	0.068	0.107
Doing nothing	rho	−0.514	−0.532	−0.321	−0.525	−0.535	−0.425
	p	0.000	0.000	0.001	0.000	0.000	0.000
Taking in pharmaceuticals	rho	−0.011	−0.071	0.034	−0.107	0.033	0.008
	p	0.916	0.483	0.734	0.289	0.743	0.939
Sense of humor	rho	0.235	0.249	0.361	0.154	0.182	0.182
	p	0.019	0.013	0.000	0.126	0.069	0.071
Perceived stress level	rho	−0.433	−0.488	−0.415	−0.476	−0.491	−0.347
	p	0.000	0.000	0.000	0.000	0.000	0.000
Acceptance of illness	rho	0.752	0.619	0.499	0.639	0.604	0.582
	p	0.000	0.000	0.000	0.000	0.000	0.000

rho—Spearman’s rho coefficient; p—test probability.

**Table 3:** Relation between perceived stress level and stress coping strategies.

Variable	Perceived Stress Level	
	rho	p
Active stress coping methods	−0.352	0.000
Planning	−0.410	0.000
Seeking instrumental support	−0.148	0.143
Seeking emotional support	−0.269	0.007
Self-blaming	0.250	0.012
Turning to religion	0.023	0.817
Positive re-evaluation	−0.394	0.000
Giving vent to one’s feeling	0.435	0.000
Acceptance	−0.240	0.016
Denial	0.303	0.002
Dealing with other things	0.011	0.914
Doing nothing	0.593	0.000
Taking in pharmaceuticals	0.195	0.052
Sense of humor	−0.354	0.000

rho—Spearman’s rho coefficient; p—test probability.

## Data Availability

Not applicable.
